# Photographic grading to evaluate facial cleanliness and trachoma among children in Amhara region, Ethiopia

**DOI:** 10.1371/journal.pntd.0012257

**Published:** 2024-07-11

**Authors:** Ramoncito L. Caleon, Fisseha Admassu, Solomon Aragie, Dagnachew Hailu, Adane Dagnew, Taye Zeru, Dionna M. Wittberg, Isabel J. B. Thompson, Seid Abdu, Social Beyecha, Tibebe Birhanu, Habib Getachew, Banchalam Getnet, Endale Kabtu, Meskerem Shibiru, Solomon Tekew, Bilen Wondimteka, Thomas M. Lietman, Scott D. Nash, Matthew C. Freeman, Jeremy D. Keenan

**Affiliations:** 1 Department of Ophthalmology, Emory University School of Medicine, Atlanta, Georgia, United States of America; 2 Department of Ophthalmology, University of Gondar, Gondar, Ethiopia; 3 The Carter Center Ethiopia, Addis Ababa, Ethiopia; 4 Francis I Proctor Foundation, University of California, San Francisco, California, United States of America; 5 Amhara Public Health Institute, Bahir Dar, Ethiopia; 6 Department of Ophthalmology, University of California, San Francisco, California, United States of America; 7 The Carter Center, Atlanta, Georgia, United States of America; 8 Department of Environmental Health, Rollins School of Public Health, Emory University, Atlanta, Georgia, United States of America; Institut Pasteur, FRANCE

## Abstract

**Background:**

Promotion of facial cleanliness is recommended for the elimination of blinding trachoma, largely because of observational studies that have found an association between various measures of facial uncleanliness and trachoma. However, when a field grader assesses both facial cleanliness and trachoma, associations may be biased. Assessment of photographs of the face and conjunctiva by masked graders may provide a less biased estimate of the relationship between facial cleanliness and trachoma.

**Methods:**

Face photographs, conjunctival photographs, and conjunctival swabs were obtained on a random sample of 0–9-year-old children from each of 40 communities in Amhara region, Ethiopia. Face photographs were assessed for the presence of seven measures of an unclean face (i.e., wet nasal discharge, dry nasal discharge, wet ocular discharge, dry ocular discharge, food, dust/dirt, and flies) by three independent masked photo-graders. Conjunctival photographs were similarly graded in a masked fashion for signs of clinically active trachoma. Conjunctival swabs were processed for *Chlamydia trachomatis* DNA.

**Results:**

Of 2073 children with complete data, 808 (39%) had evidence of clinically active trachoma, 150 (7%) had evidence of ocular chlamydia infection, and 2524 (91%) had at least one measure of an unclean face. Dry ocular discharge had the strongest association with clinically active trachoma (age- and sex-adjusted prevalence ratio [PR] 1.4, 95% CI 1.2–1.6) and ocular chlamydia infection (PR 1.9, 95%CI 1.3–2.9), although significant associations were observed between each of the measures of facial uncleanliness and trachoma.

**Conclusions:**

Masked assessment of face and conjunctival photographs confirmed prior observational studies that have noted associations between various measures of facial uncleanliness and trachoma. The causal relationship between facial uncleanliness and trachoma is unclear since many features used to measure facial cleanliness (e.g., ocular discharge, nasal discharge, and flies) could be consequences of antecedent ocular chlamydia infection.

**Trial registration:**

NCT02754583, clinicaltrials.gov.

## Author summary

We graded face and eye photographs from children in Ethiopia to characterize the relationship between seven measures of an unclean face and several trachoma outcomes. Dry ocular discharge had the strongest associations with trachoma outcomes, and there was a dose-dependent relationship between the seven facial uncleanliness measures and trachoma. Moreover, using a composite measure of facial uncleanliness, such as the presence of nasal discharge, ocular discharge, or flies, had even stronger associations with trachoma. However, it is difficult to determine the importance of these measures of facial uncleanliness as risk factors for trachoma since ocular chlamydia infection can cause ocular discharge and potentially nasal discharge, each of which may in turn attract flies.

## Introduction

Trachoma is the most common infectious cause of blindness, affecting approximately two million people worldwide [1,2]. The WHO has endorsed the four-pronged SAFE strategy for the elimination of trachoma: Surgery for trichiasis, Antibiotics, Facial cleanliness, and Environmental improvements in water, sanitation, and hygiene (WASH) [3]. Facial cleanliness is thought to be important for clearing the infectious agent, *Chlamydia trachomatis*, which is present in ocular and nasal secretions, and for reducing face flies, which may serve as a mechanical vector of chlamydia [4,5]. Observational studies have frequently found an association between various features of an unclean face and the clinical signs of trachoma [6]. However, in most prior studies the face and conjunctiva have been evaluated at the same time by the same person. Such a study design may have introduced bias since it is possible that an examiner’s assessment of the face findings could be influenced by the conjunctiva, and vice versa.

In a recent study, we performed face photography and conjunctival photography in communities with trachoma. We graded the photographs for facial cleanliness and trachoma in a masked fashion, providing an opportunity to assess the relationship between facial cleanliness and trachoma in a less biased way. Using this more objective and rigorous method, the goal of the present analysis was to determine which measures of an unclean face were most associated with clinically active trachoma and ocular chlamydial infection.

## Methods

### Ethics statement

Study protocols were approved by human subjects review boards at the University of California, San Francisco; Emory University; the Food, Medicine, and Health Care Administration and Control Authority of Ethiopia; and the Ethiopian Ministry of Science and Technology. Due to high illiteracy levels in the study area, verbal consent was obtained from the participant’s guardians.

### Study design and participants

The present study is an observational analysis using baseline data from the WASH Upgrades for Health in Amhara trial (WUHA; clinicaltrials.gov NCT02754583) collected between November 9, 2015 and April 16, 2016 [7,8]. WUHA was a cluster-randomized trial performed in a trachoma-hyperendemic region of Ethiopia that assessed the effectiveness of an integrated WASH intervention for control of trachoma. Prior to randomization a door-to-door population census was performed in each of 40 study communities, and then a stratified random sample of approximately 30 children aged 0–5 years and 30 children aged 6–9 years per community was assessed for facial cleanliness and trachoma.

### Procedures

Photographs of the face and the everted right superior tarsal conjunctiva were taken in triplicate with a Samsung Galaxy NX camera equipped with a 60 mm ƒ/2.8 macro lens (Seoul, South Korea) using the native flash and the following camera settings: aperture priority (ƒ/11 for face and ƒ/32 for conjunctiva), ISO 400, and automatic white balance. For face photographs, flies were shooed away with a hand one time only, and photographs taken a few seconds thereafter. A Dacron swab (Puritan Medical Products, Guildford, ME, USA) was then passed three times over the everted conjunctiva. Conjunctival swabs were stored at -20°C and processed at the Amhara Public Health Institute (Bahir Dar, Ethiopia) with the Abbott RealTime assay (Abbott Molecular, Des Plaines, IL, USA) on the m2000 platform to detect *C*. *trachomatis* DNA. Swabs were initially processed with PCR in pools of five randomly selected swabs per age stratum per community. For the 0–5 year-old age group, each of the individual swabs from a positive pool was subsequently processed with PCR. For the 6–9 year-old age group, individual swabs were processed only from positive pools in those study clusters in which more than 80% of pools were positive.

### Photographic grading

Face and conjunctival photographs were graded by three two-person teams of trained ophthalmology residents at the Gondar Grading Center, which is housed at the University of Gondar. Graders received a three-day training for assessment of trachomatous inflammation—follicular (TF) and trachomatous inflammation—intense (TI) according to the World Health Organization’s simplified grading system [9]. Trainees were required to achieve sufficient agreement with an experienced grader (JDK) before being certified to grade, defined as a Cohen’s kappa of 0.7 or greater on a set of 50 photographs. When grading face photographs, the graders were masked to the results of conjunctival photo-grading, and vice versa. Graders were not provided with any accompanying information (e.g., participant identifier, village identifier, time point). Face photographs were evaluated for the presence of each of the following seven features: wet nasal discharge, dry nasal discharge, wet ocular discharge, dry ocular discharge, food, dust/dirt, and flies. Nasal discharge was defined as discharge running from the nares along the upper lip. Ocular discharge was defined as discharge touching the eyelid margin, eyelashes, or corners of the eye. Whether discharge was wet or dry was judged according to the opinion of the grader based on their experience as well as training photographs. Food, dust, and dirt were graded on the entire face (i.e., forehead to chin, ear to ear). The presence of food was judged according to the opinion of the grader, acknowledging that food would typically be present near the mouth and have a different color than dirt/dust. The seven features were graded all at the same time without any attempt to grade the different facial cleanliness indicators independently. Photographs were taken in triplicate to increase the chances of a high-quality image. Photographs from each child-visit were presented as a set of three conjunctival images or three face images, and a single grade was provided for that set of images. Each of the three teams provided a grade for each photograph set in the study, and the majority consensus of the three grades was used for analyses.

### Data analysis

**Fig 1:**
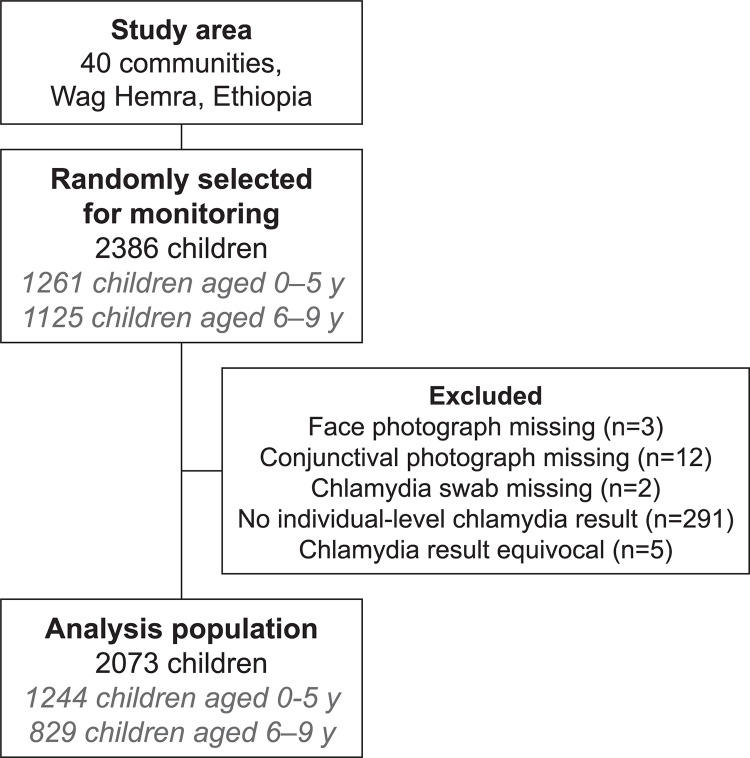
Participant flow. A stratified random sample of approximately 30 children aged 0–5 years and 30 children aged 6–9 years from each of 40 communities in Ethiopia was selected for face photography, conjunctival photography, and conjunctival swabbing.

The analysis population consisted of 2073 children aged 0–9 years with complete data for face photographs, conjunctival photographs, and ocular chlamydia PCR, from a total of 2386 children randomly selected for monitoring (Fig 1). The four outcome variables for this study included TF, TI, clinically active trachoma (defined as TF and/or TI), and ocular chlamydia. The exposure variables included the presence of each of the measures of an unclean face, various composite measures of an unclean face, and a facial uncleanliness summary score, calculated as the sum of the seven individual binary measures (range: 0 to 7). Inter-rater agreement was estimated for the three photo-graders with Gwet’s AC1. The association between each trachoma outcome and each measure of an unclean face was assessed as a prevalence ratio adjusted for age and sex in a Poisson regression with a Huber-White sandwich variance estimator to account for intragroup correlation of study clusters [10]. Statistical analyses were conducted using R v4.3 (R Project for Statistical Computing, Vienna, Austria).

## Results

Of 2073 children with complete data, the median age was 5 years (interquartile range 2–7) and 1068 (52%) were female. Inter-rater agreement between the three photo-grading teams for the features of facial uncleanliness was moderate or better for most of the features, with a Gwet’s AC1 ranging from 0.32 (95% CI 0.29–0.35) for dust/dirt to 0.84 (95% CI 0.82–0.86) for flies (Table 1). Subsequent analyses used the majority consensus of the three grades.

**Table 1:** Inter-rater agreement between three teams of photo-graders for seven measures of an unclean face.

Variable	Gwet’s AC1 (95% CI)
Wet nasal discharge	0.49 (0.46–0.52)
Dry nasal discharge	0.48 (0.46–0.51)
Wet ocular discharge	0.63 (0.60–0.65)
Dry ocular discharge	0.32 (0.28–0.35)
Food	0.36 (0.33–0.39)
Dirt	0.32 (0.29–0.35)
≥ 1 fly	0.84 (0.82–0.86)

**Fig 2:**
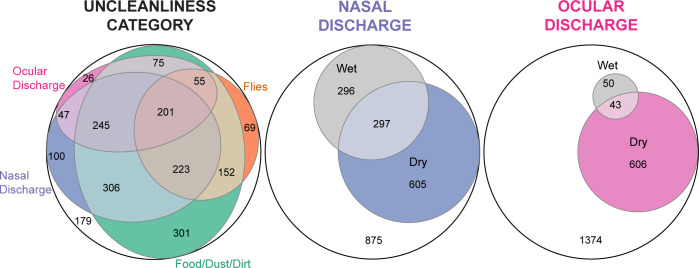
Overlap between various components of an unclean face. Area-proportional Venn diagrams show the overlap in uncleanliness measures in a given child. The left panel depicts the overlap for (i) nasal discharge—either wet or dry, (ii) ocular discharge—either wet or dry, (iii) food or dust/dirt, and (iv) flies on the face; the middle panel depicts the overlap for wet and dry nasal discharge; and the right panel depicts the overlap for wet and dry ocular discharge.

The presence of dust/dirt was the most prevalent feature of facial uncleanliness (76%) and wet ocular discharge was the least prevalent (4%) (Table 2). If an unclean face was classified as any nasal discharge, any ocular discharge, or flies, then 77% of children were classified as having an unclean face. When defined as any nasal discharge, any ocular discharge, flies, or dirt, then 91% of children were classified as having an unclean face. The mean seven-point facial uncleanliness indicator total was 2.3 (95% CI 2.2–2.4). While there was considerable overlap of various features of facial uncleanliness, none appeared to be redundant (Fig 2).

**Table 2:** Characteristics of the study population. Values for face photographs and conjunctiva photographs indicate the presence of each feature using the majority consensus from three independent photo-graders. Ocular chlamydia was assessed by polymerase chain reaction from conjunctival swabs. Bootstrapped 95% confidence intervals were resampled at the community level to account for intracluster correlation (n = 999 replications).

Variable	Number (n = 2703)	Proportion (95% CI)
**Face photographs**		
Wet nasal discharge	593	29% (26–31%)
Dry nasal discharge	902	44% (40–47%)
Wet ocular discharge	93	4% (3–6%)
Dry ocular discharge	649	31% (27–36%)
Food	88	4% (3–5%)
Dust/dirt	1554	75% (72–78%)
≥ 1 fly	794	38% (33–45%)
Nasal or ocular discharge or ≥ 1 fly	1593	77% (72–81%)
Nasal or ocular discharge or dirt or ≥ 1 fly	1894	91% (89–93%)
No. of facial uncleanliness features		
0	179	9% (7–10%)
1	472	23% (20–26%)
2	548	26% (24–29%)
3	489	24% (21–26%)
4	297	14% (12–17%)
5	78	4% (3–5%)
6	10	<1% (0–1%)
7	0	0%
**Conjunctiva photographs**		
TF	619	30% (26–33%)
TI	384	19% (16–22%)
TF and/or TI	808	39% (35–43%)
**Conjunctival swabs**		
*Chlamydia trachomatis*	150	7% (5–10%)

TF, trachomatous inflammation–follicular; TI, trachomatous inflammation–intense.

**Fig 3:**
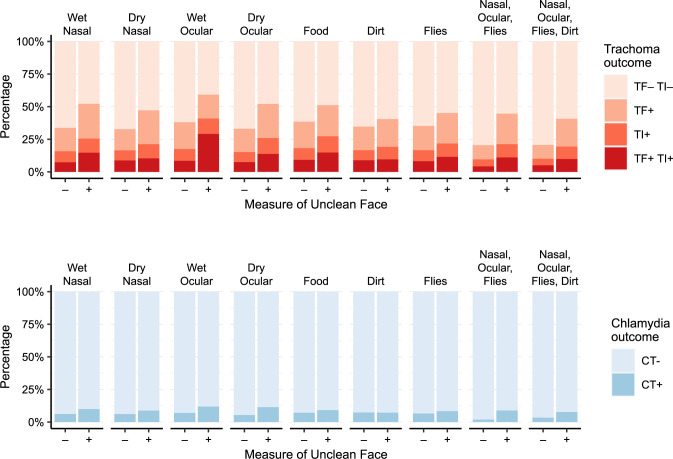
Prevalence of trachoma outcomes stratified by various measures of an unclean face. Each pair of bars represents the study population stratified by the presence (+) or absence (–) of a measure of facial uncleanliness, with the seven individual measures on the left and two composite measures on the right. Clinical trachoma outcomes are shown in the top panel and ocular chlamydia infection in the bottom panel.

TF was present in 30% of participants, TI in 19%, TF and/or TI in 39%, and ocular chlamydia in 7% (Table 2). Trachoma indicators were more prevalent among children with each of the seven features of facial uncleanliness (Fig 3). Clinically active trachoma (i.e., the presence of TF and/or TI) was most common in those with wet ocular discharge (59%, 95% CI 47–71%), followed by wet nasal discharge (52%, 95% CI 46–59%) and dry ocular discharge (52%, 95% CI 46–58%). Ocular chlamydia was most prevalent in those with wet ocular discharge (12%, 95% CI 4–21%), followed by dry ocular discharge (11%, 95% CI 7–17%) and wet nasal discharge (10%, 95% CI 6–14%).

**Fig 4:**
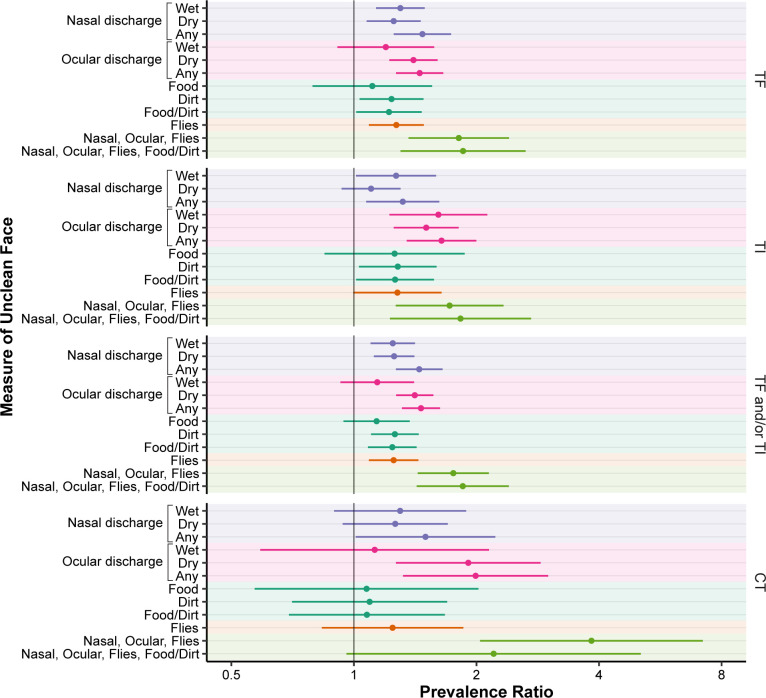
Association between measures of facial uncleanliness and trachoma. The points and bars represent age- and sex-adjusted prevalence ratios for each trachoma outcome and 95% confidence intervals. The numeric results used to make the figure are provided in S1 Table. Abbreviations: TF, trachomatous inflammation–follicular; TI, trachomatous inflammation–intense; CT, ocular *C*. *trachomatis*.

Associations between each of the seven measures of facial uncleanliness and trachoma were expressed as prevalence ratios of the association between the facial uncleanliness feature (i.e., exposure) and the trachoma indicator (i.e., outcome) (Fig 4). The measure of facial uncleanliness most associated with clinically active trachoma (i.e., TF and/or TI) was dry ocular discharge (age- and sex-adjusted prevalence ratio [PR] 1.4, 95% CI 1.3–1.6), followed by dirt on the face (PR 1.3, 95% CI 1.1–1.4). The magnitude of association was greater for various composite measures, including (i) the presence of any nasal discharge, any ocular discharge, flies, or dirt on the face (PR 1.9, 95%CI 1.4–2.4); (ii) the presence of any nasal discharge, any ocular discharge, or flies on the face (PR 1.8, 95%CI 1.4–2.1); (iii) any ocular discharge (PR 1.5, 95%CI 1.3–1.6); and (iv) any nasal discharge (PR 1.4, 95%CI 1.3–1.7). Results had a similar pattern for the ocular chlamydia outcome, although with wider confidence intervals and a greater magnitude of association for some of the composite outcomes (e.g., presence of any nasal discharge, any ocular discharge, or flies had a PR of 3.8, 95%CI 2.0–7.2; the presence of any ocular discharge had a PR of 2.0, 95%CI 1.3–3.0). Associations were attenuated in multivariable models that adjusted for all measures of facial uncleanliness, with the strongest independent associations observed for dry ocular discharge, wet nasal discharge, and flies on the face (S1 Fig).

**Fig 5:**
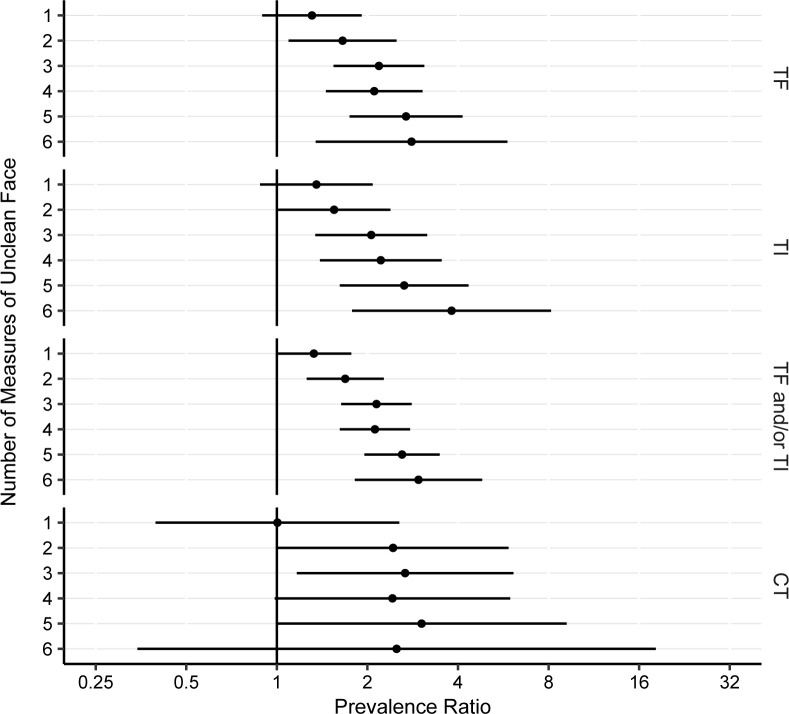
The association between trachoma outcomes and the total number of facial uncleanliness measures. The reference value was a score of 0 (i.e., absence of all facial uncleanliness features). The numeric results used to make the figure are provided in S2 Table. Abbreviations: TF, trachomatous inflammation–follicular; TI, trachomatous inflammation–intense; CT, ocular *C*. *trachomatis*.

Children with more features of facial uncleanliness were more likely to have clinical trachoma, with a monotonic increase in the PR for each additional point increase in the seven-point facial uncleanliness indicator total (Fig 5). For example, the PR for clinically active trachoma (i.e., TF and/or TI) went from 1.3 (95% CI 1.0–1.8) when a single feature of facial uncleanliness was present, to 3.0 (95% CI 1.8–4.8) when 6 features were present. A monotonic trend was less apparent for the ocular chlamydia outcome, with a similar magnitude of association for each additional facial uncleanliness measures once at least two measures were present (Fig 5).

## Discussion

Masked assessment of face and conjunctival photographs was used in the present study to estimate the association between various features of facial uncleanliness and trachoma. The masked approach should have resulted in less biased estimates compared with prior studies that have had the same field grader evaluate the face and conjunctiva at the same time. Facial uncleanliness was very common in the study population, with approximately two-thirds of children having two or more features of facial uncleanliness. The single feature most associated with both clinically active trachoma and ocular chlamydia infection was the presence of dry ocular discharge. Several composite outcomes had even stronger associations with trachoma outcomes, such as the presence of ocular discharge, nasal discharge, or flies. Children with a greater number of features of facial uncleanliness were more likely to have clinical trachoma.

To our knowledge, only one prior study has relied on face photographs graded by trained image graders to evaluate facial cleanliness [11]. Consistent with this prior study we found variable agreement between masked photo-graders when examining several features of facial uncleanliness on photographs—lower than some prior studies using field graders [12–14]. The low inter-rater agreement for some of the features of facial uncleanliness suggests grading photographs for facial uncleanliness may be an inherently noisy test, likely due to the subjective nature of the exercise. Nonetheless, there may be ways to improve precision, including deep learning approaches as well as aggregating multiple independent assessments.

**Fig 6:**
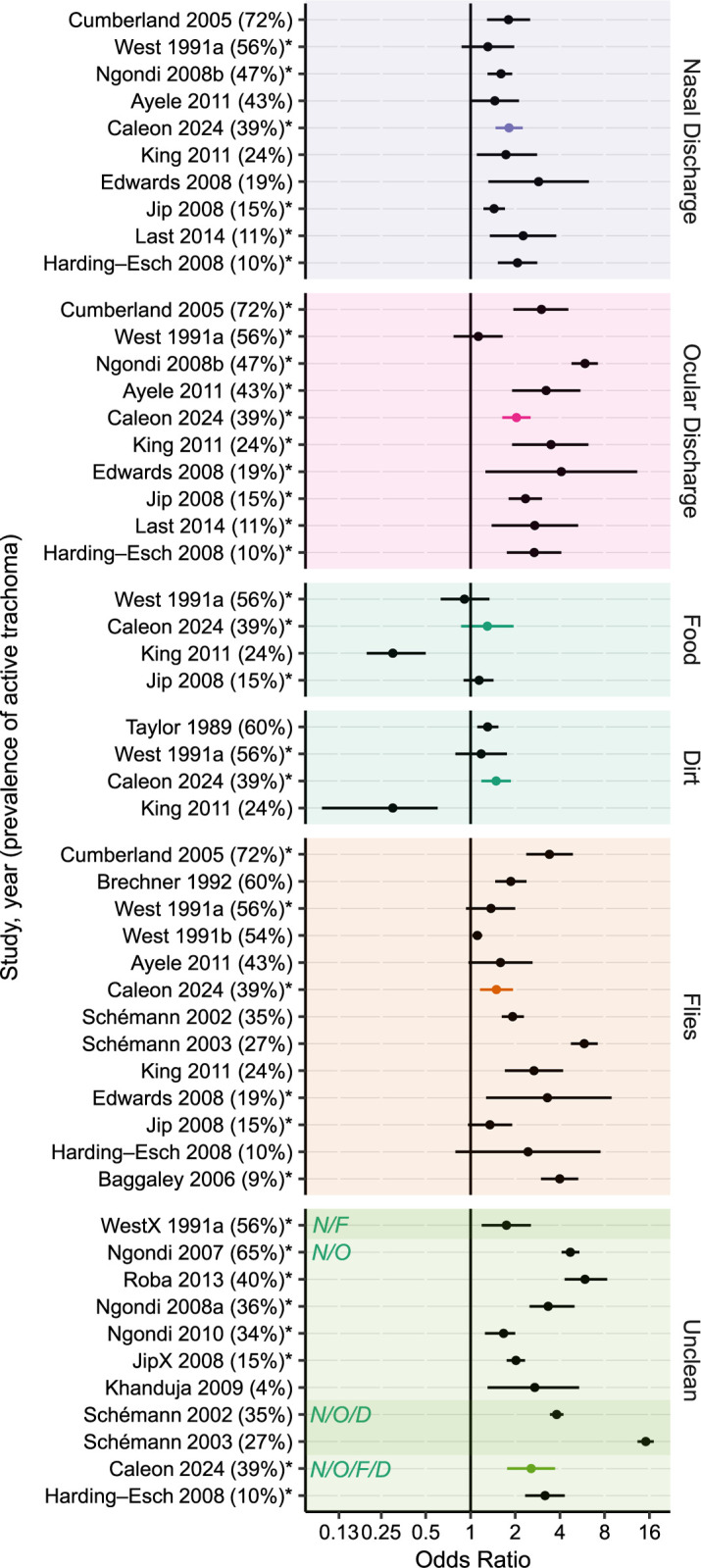
Association between measures of an unclean face with clinically active trachoma (i.e., TF and/or TI) in selected studies. Points and bars represent odds ratios and 95% confidence intervals. Measures of association for the present study were re-calculated with logistic regression to improve comparability with other studies (S3 Table). Studies are listed in descending order of trachoma prevalence (provided in parentheses). Estimates produced by multivariable models are indicated with an asterisk. Studies assessing composite measures used different definitions of an unclean face, which are indicated in the shaded boxes (N/F = nasal discharge or flies on face; N/O = nasal or ocular discharge; N/O/D = nasal discharge, ocular discharge, or dirt; N/O/F/D = nasal discharge, ocular discharge, flies, or dirt).

**Fig 7:**
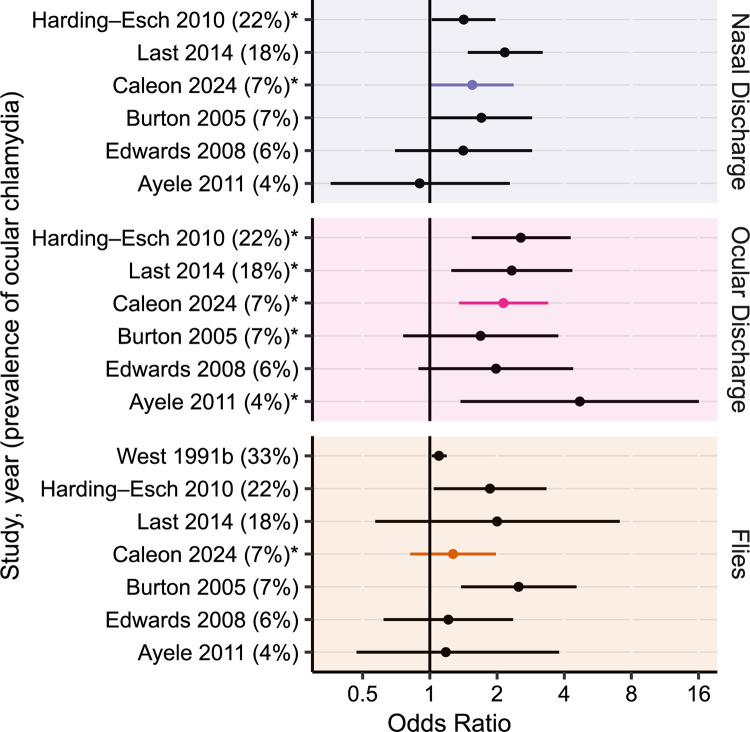
Association between measures of an unclean face and ocular chlamydia in selected studies. Points and bars represent odds ratios and 95% confidence intervals. Measures of association for the present study were re-calculated with logistic regression to improve comparability with other studies (S3 Table). Studies are listed in descending order of ocular chlamydia prevalence (provided in parentheses). Estimates produced by multivariable models are indicated with an asterisk.

The magnitude of the associations found in this study were generally lower than previously reported (Figs 6 and 7) [6,11,13,15–36]. Several factors could account for this difference. Face photo-graders in the present study were masked to the results of trachoma grading, and also masked to the living conditions of participants and their family members. Thus it is possible that this masked assessment reduced unconscious biases that may be present when grading in the field, resulting in weaker associations. In addition, the present study used a prevalence ratio as the measurement of association. Prior studies have typically estimated the association between facial uncleanliness and trachoma with odds ratios, which may have resulted in overestimates in places where trachoma was not a rare event [10,37]. Alternatively, it is possible that facial uncleanliness is less important in areas with hyperendemic trachoma—including the study area of the present report—where the force of infection may overpower any individual-level hygiene risk factors.

Differentiating between wet and dry discharge allowed us to individually analyze 7 facial components as well as a seven-point facial uncleanliness summary score to further characterize facial cleanliness. We found that the observed measures of an unclean face exhibited a dose-dependent relationship with clinical trachoma outcomes, with a higher prevalence of trachoma in children with more features of facial uncleanliness. While few studies have explored this dose-dependent relationship, our results are consistent with one prior study in which the presence of ocular discharge and face flies had a greater association with TF and/or TI than did ocular discharge alone [31].

Measures of facial uncleanliness could be used to assess the effectiveness of a face washing program by indicating if a child recently washed their face. In this case, the presence of dirt or food might be a logical choice as an indicator, and would be supported by this study’s finding of an association between food/dirt and clinically active trachoma. A prior study that randomized children to face washing or no face washing and then assessed facial cleanliness found that lack of ocular discharge and lack of dry nasal discharge were most indicative of a recently washed face [11].

This study found that most measures of facial uncleanliness had weaker associations with ocular chlamydia than with the clinical signs of trachoma, although the general patterns were similar. Composite measures of facial uncleanliness (e.g., nasal discharge, ocular discharge, or flies) had a much stronger association with ocular chlamydia than any of the individual measures. Most previous studies have failed to demonstrate a significant association between facial uncleanliness and ocular chlamydia, although a 2014 meta-analysis found that ocular and nasal discharge were significantly associated with ocular chlamydia after pooling the results of four studies [6].

It is unclear which features of facial uncleanliness are most appropriate to define an unclean face in the context of trachoma. If based solely on the strength of association between facial uncleanliness and trachoma, then ocular discharge might be proposed as an important measure of facial uncleanliness for trachoma programs—a finding consistent with prior studies (Figs 6 and 7). However, ocular discharge is a direct consequence of ocular chlamydia infection and thus likelier to be a marker of prior chlamydia infection rather than a risk factor for future infection. The utility of such a marker is unclear when better indicators of chlamydial infection (i.e., polymerase chain reaction tests) and clinically active trachoma (i.e., conjunctival examination) are available. Nasal discharge and face flies might also be considered for defining an unclean face given the findings of this study and prior studies (Figs 6 and 7). Each of these has been postulated as potential transmission routes for ocular chlamydia infection [13,38]. However, it is unclear whether chlamydia found in the nares represents a true nasopharyngeal chlamydia infection that can occur in the absence of a concomitant ocular infection, or instead is simply the result of nasolacrimal drainage from ocular chlamydia infection [38,39]. Flies may conceptually have a clearer role as a cause of chlamydia infection, but even here it is possible that flies are merely attracted to eyes with ocular secretions from ocular chlamydial infection, and are not actually a major part of the causal pathway. Aside from the possibility that trachoma itself may cause facial features traditionally considered part of an unclean face, it is also possible that facial uncleanliness may be a confounder along the causal pathway, associated with some other exposure (e.g., unclean hands, unclean clothes) that causes trachoma. Indeed, the causality is difficult to tease out, making these indicators of facial uncleanliness problematic when used as risk factors for subsequent transmission of trachoma. Based on the relatively weak associations found in this study, as well as the challenges in interpreting the causal pathway of these associations, it may not be worthwhile for most trachoma programs to incorporate facial cleanliness assessments into trachoma surveys.

A number of limitations must be acknowledged when interpreting this study. First, despite the masked approach of photo-grading, the graders could still be subject to bias based on the apparent age or gender of the participant. Moreover, while conjunctival and face photographs were graded independently, the seven features of facial uncleanliness were not. It is possible that the presence of one feature of facial uncleanliness may have made a grader more likely to find a different feature. Second, we did not conduct in-field facial cleanliness assessments to compare with our photographic grades. A systematic review found acceptable agreement between grades from conjunctival photography versus trachoma grades in the field, but similar studies for face photographs are lacking [40]. Third, the study was not designed to assess the relationship between measures of facial uncleanliness and face-washing behavior. Fourth, the results may depend on the specific methods used in this study, including the cameras and photograph protocols. For example, we shooed flies away prior to the photograph to increase the specificity of this finding, but this may have reduced the prevalence of face flies observed in this population. Finally, the observed associations may be sensitive to the prevalence of trachoma since it is conceivable that any individual-level risk factors may be less important when the community burden of infection is high. The generalizability to areas with less prevalent trachoma, or where hygiene and eating practices differ, is unclear.

In summary, the present study used masked photo-graders to determine the association between several measures of facial uncleanliness and trachoma in a less biased fashion. The study found a dose-dependent relationship between seven measures of an unclean face and trachoma outcomes. Ocular discharge, and especially dry ocular discharge, was most strongly associated with clinically active trachoma and ocular chlamydia infection, but other measures of uncleanliness such as nasal discharge, flies on the face, and dirt on the face were also associated with trachoma outcomes—albeit with lower magnitudes of association compared with many prior studies.

## Supporting information

S1 Table Association between measures of facial uncleanliness and trachoma outcomes. Estimates represent the age- and sex-adjusted prevalence ratio (PR) and 95% confidence interval (CI) assessing the relationship between individual measures of facial uncleanliness and each of the four trachoma outcomes. Values are graphically depicted in Fig 4.
(DOCX)

S2 Table Association between number of measures of facial uncleanliness and trachoma outcomes. Estimates represent the age- and sex-adjusted prevalence ratio (PR) and 95% confidence interval (CI) assessing the relationship between the number of measures of facial uncleanliness (i.e., 1 through 6, assessed relative to zero measures of facial uncleanliness) and each of the four trachoma outcomes. Values are graphically depicted in Fig 5.
(DOCX)

S3 Table Odds ratios between selected measures of facial uncleanliness and trachoma outcomes. Estimates represent the age- and sex-adjusted odds ratio (OR) and 95% confidence interval (CI) assessing the relationship between individual measures of facial uncleanliness and two trachoma outcomes: (i) trachomatous inflammation–follicular (TF) and/or trachomatous inflammation–intense (TI), and (ii) ocular *Chlamydia trachomatis* (CT) infection. Selected values are graphically depicted in Figs 6 and 7.
(DOCX)

S1 Fig Association between measures of facial uncleanliness and trachoma outcomes when adjusting for each of the individual measures of facial uncleanliness. The points and bars in the plot represent age- and sex-adjusted prevalence ratios for each trachoma outcome and 95% confidence intervals. The numeric results used to make the figure are provided below. Abbreviations: TF, trachomatous inflammation–follicular; TI, trachomatous inflammation–intense; CT, ocular *C*. *trachomatis*.
(DOCX)

S1 Data Study data. Demographic information and the presence or absence of each measure of trachoma and facial uncleanliness is provided for each child.
(CSV)
