# Supplementary material for: Photographic grading to evaluate facial cleanliness and trachoma among children in Amhara region, Ethiopia
Source: PLoS Negl Trop Dis. 2024 Jul 11;18(7):e0012257. doi: 10.1371/journal.pntd.0012257 (PMC11290635; doi:10.1371/journal.pntd.0012257)
Supplement: S1 Table — Estimates represent the age- and sex-adjusted prevalence ratio (PR) and 95% confidence interval (CI) assessing the relationship between individual measures of facial uncleanliness and each of the four trachoma outcomes. Values are graphically depicted in Fig 4. (DOCX) [file pntd.0012257.s001.docx]

**S1 Table. Association between measures of facial uncleanliness and trachoma outcomes.** Estimates represent the age- and sex-adjusted prevalence ratio (PR) and 95% confidence interval (CI) assessing the relationship between individual measures of facial uncleanliness and each of the four trachoma outcomes. Values are graphically depicted in Fig 4.

|  | **TF** |  |  | **TI** |  |  | **TF and/or TI** |  |  | **CT** |  |
| --- | --- | --- | --- | --- | --- | --- | --- | --- | --- | --- | --- |
| **Facial uncleanliness measure** | **PR (95%CI)** | ***P*-value** |  | **PR (95%CI)** | ***P*-value** |  | **PR (95%CI)** | ***P*-value** |  | **PR (95%CI)** | ***P*-value** |
| **Individual measures** |  |  |  |  |  |  |  |  |  |  |  |
| Wet nasal discharge | 1.3 (1.1–1.5) | <0.001 |  | 1.3 (1.0–1.6) | 0.039 |  | 1.2 (1.1–1.4) | 0.001 |  | 1.3 (0.9–1.9) | 0.170 |
| Dry nasal discharge | 1.3 (1.1–1.5) | 0.004 |  | 1.1 (0.9–1.3) | 0.254 |  | 1.3 (1.1–1.4) | <0.001 |  | 1.3 (0.9–1.7) | 0.123 |
| Wet ocular discharge | 1.2 (0.9–1.6) | 0.196 |  | 1.6 (1.2–2.1) | 0.001 |  | 1.1 (0.9–1.4) | 0.214 |  | 1.1 (0.6–2.1) | 0.721 |
| Dry ocular discharge | 1.4 (1.2–1.6) | <0.001 |  | 1.5 (1.3–1.8) | <0.001 |  | 1.4 (1.3–1.6) | <0.001 |  | 1.9 (1.3–2.9) | 0.002 |
| Food | 1.1 (0.8–1.6) | 0.546 |  | 1.3 (0.8–1.9) | 0.255 |  | 1.1 (0.9–1.4) | 0.179 |  | 1.1 (0.6–2.0) | 0.824 |
| Dirt | 1.2 (1.0–1.5) | 0.021 |  | 1.3 (1.0–1.6) | 0.027 |  | 1.3 (1.1–1.4) | 0.001 |  | 1.1 (0.7–1.7) | 0.691 |
| Flies | 1.3 (1.1–1.5) | 0.002 |  | 1.3 (1.0–1.6) | 0.053 |  | 1.3 (1.1–1.4) | 0.002 |  | 1.2 (0.8–1.9) | 0.284 |
| **Composite measures (≥ 1 of the following)** |  |  |  |  |  |  |  |  |  |  |  |
| Any nasal discharge (wet and/or dry) | 1.5 (1.3–1.7) | <0.001 |  | 1.3 (1.1–1.6) | 0.009 |  | 1.4 (1.3–1.7) | <0.001 |  | 1.5 (1.0–2.2) | 0.044 |
| Any ocular discharge (wet and/or dry) | 1.5 (1.3–1.7) | <0.001 |  | 1.6 (1.3–2.0) | <0.001 |  | 1.5 (1.3–1.6) | <0.001 |  | 2.0 (1.3–3.0) | 0.001 |
| Food, dirt | 1.2 (1.0–1.5) | 0.036 |  | 1.3 (1.0–1.6) | 0.038 |  | 1.2 (1.1–1.4) | 0.002 |  | 1.1 (0.7–1.7) | 0.744 |
| Any nasal, any ocular, flies | 1.8 (1.4–2.4) | <0.001 |  | 1.7 (1.3–2.3) | 0.001 |  | 1.8 (1.4–2.1) | <0.001 |  | 3.8 (2.0–7.2) | <0.001 |
| Any nasal, any ocular, flies, food, dirt | 1.9 (1.3–2.6) | 0.001 |  | 1.8 (1.2–2.7) | 0.003 |  | 1.9 (1.4–2.4) | <0.001 |  | 2.2 (1.0–5.1) | 0.063 |

CT = ocular *Chlamydia trachomatis*; TF = trachomatous inflammation–follicular; TI = trachomatous inflammation–intense
