# Supplementary material for: Photographic grading to evaluate facial cleanliness and trachoma among children in Amhara region, Ethiopia
Source: PLoS Negl Trop Dis. 2024 Jul 11;18(7):e0012257. doi: 10.1371/journal.pntd.0012257 (PMC11290635; doi:10.1371/journal.pntd.0012257)
Supplement: S2 Table — Estimates represent the age- and sex-adjusted prevalence ratio (PR) and 95% confidence interval (CI) assessing the relationship between the number of measures of facial uncleanliness (i.e., 1 through 6, assessed relative to zero measures of facial uncleanliness) and each of the four trachoma outcomes. Values are graphically depicted in Fig 5. (DOCX) [file pntd.0012257.s002.docx]

**S2 Table. Association between number of measures of facial uncleanliness and trachoma outcomes.** Estimates represent the age- and sex-adjusted prevalence ratio (PR) and 95% confidence interval (CI) assessing the relationship between the number of measures of facial uncleanliness (i.e., 1 through 6, assessed relative to zero measures of facial uncleanliness) and each of the four trachoma outcomes. Values are graphically depicted in Fig 5.

|  | **TF** |  |  | **TI** |  |  | **TF and/or TI** |  |  | **CT** |  |
| --- | --- | --- | --- | --- | --- | --- | --- | --- | --- | --- | --- |
| **No** | **PR (95%CI)** | ***P*-value** |  | **PR (95%CI)** | ***P*-value** |  | **PR (95%CI)** | ***P*-value** |  | **PR (95%CI)** | ***P*-value** |
| 1 | 1.3 (0.9–1.9) | 0.167 |  | 1.4 (0.9–2.1) | 0.169 |  | 1.3 (1.0–1.8) | 0.053 |  | 1.0 (0.4–2.6) | 0.993 |
| 2 | 1.7 (1.1–2.5) | 0.017 |  | 1.5 (1.0–2.4) | 0.046 |  | 1.7 (1.3–2.3) | 0.001 |  | 2.4 (1.0–5.9) | 0.048 |
| 3 | 2.2 (1.5–3.1) | <0.001 |  | 2.1 (1.3–3.2) | 0.001 |  | 2.1 (1.6–2.8) | <0.001 |  | 2.7 (1.2–6.1) | 0.020 |
| 4 | 2.1 (1.5–3.0) | <0.001 |  | 2.2 (1.4–3.5) | 0.001 |  | 2.1 (1.6–2.8) | <0.001 |  | 2.4 (1.0–6.0) | 0.054 |
| 5 | 2.7 (1.7–4.1) | <0.001 |  | 2.6 (1.6–4.3) | <0.001 |  | 2.6 (2.0–3.5) | <0.001 |  | 3.0 (1.0–9.2) | 0.051 |
| 6 | 2.8 (1.3–5.8) | 0.006 |  | 3.8 (1.8–8.2) | 0.001 |  | 3.0 (1.8–4.8) | <0.001 |  | 2.5 (0.3–18.2) | 0.365 |

CT = ocular *Chlamydia trachomatis*; TF = trachomatous inflammation–follicular; TI = trachomatous inflammation–intense
