# Supplementary material for: Photographic grading to evaluate facial cleanliness and trachoma among children in Amhara region, Ethiopia
Source: PLoS Negl Trop Dis. 2024 Jul 11;18(7):e0012257. doi: 10.1371/journal.pntd.0012257 (PMC11290635; doi:10.1371/journal.pntd.0012257)
Supplement: S3 Table — Estimates represent the age- and sex-adjusted odds ratio (OR) and 95% confidence interval (CI) assessing the relationship between individual measures of facial uncleanliness and two trachoma outcomes: (i) trachomatous inflammation–follicular (TF) and/or trachomatous inflammation–intense (TI), and (ii) ocular Chlamydia trachomatis (CT) infection. Selected values are graphically depicted in Figs 6 and 7. (DOCX) [file pntd.0012257.s003.docx]

**S3 Table. Odds ratios between selected measures of facial uncleanliness and trachoma outcomes.** Estimates represent the age- and sex-adjusted odds ratio (OR) and 95% confidence interval (CI) assessing the relationship between individual measures of facial uncleanliness and two trachoma outcomes: (i) trachomatous inflammation–follicular (TF) and/or trachomatous inflammation–intense (TI), and (ii) ocular *Chlamydia trachomatis* (CT) infection. Selected values are graphically depicted in Figs 6 and 7.

|  | **TF and/or TI** |  |  | **CT** |  |
| --- | --- | --- | --- | --- | --- |
| **Facial uncleanliness measure** | **OR (95%CI)** | ***P*-value** |  | **OR (95%CI)** | ***P*-value** |
| Any nasal discharge | 1.8 (1.5–2.2) | <0.001 |  | 1.6 (1.0–2.4) | 0.04 |
| Any ocular discharge | 2.0 (1.6–2.5) | <0.001 |  | 2.1 (1.4–3.4) | 0.001 |
| Food | 1.3 (0.9–1.9) | 0.21 |  | 1.1 (0.5–2.2) | 0.82 |
| Dirt/dust | 1.5 (1.2–1.9) | <0.001 |  | 1.1 (0.7–1.8) | 0.69 |
| Flies | 1.5 (1.2–1.9) | 0.002 |  | 1.3 (0.8–2.0) | 0.29 |
| Any nasal, any ocular, flies, food, dirt | 2.6 (1.8–3.7) | <0.001 |  | 2.3 (1.0-5.6) | 0.06 |
