# Supplementary material for: Photographic grading to evaluate facial cleanliness and trachoma among children in Amhara region, Ethiopia
Source: PLoS Negl Trop Dis. 2024 Jul 11;18(7):e0012257. doi: 10.1371/journal.pntd.0012257 (PMC11290635; doi:10.1371/journal.pntd.0012257)
Supplement: S1 Fig — The points and bars in the plot represent age- and sex-adjusted prevalence ratios for each trachoma outcome and 95% confidence intervals. The numeric results used to make the figure are provided below. Abbreviations: TF, trachomatous inflammation–follicular; TI, trachomatous inflammation–intense; CT, ocular C. trachomatis. (DOCX) [file pntd.0012257.s004.docx]

**S1 Figure. Association between measures of facial uncleanliness and trachoma outcomes when adjusting for each of the individual measures of facial uncleanliness.** The points and bars in the plot represent age- and sex-adjusted prevalence ratios for each trachoma outcome and 95% confidence intervals. The numeric results used to make the figure are provided below. Abbreviations: TF, trachomatous inflammation–follicular; TI, trachomatous inflammation–intense; CT, ocular *C. trachomatis*.

|  | **TF** |  |  | **TI** |  |  | **TF and/or TI** |  |  | **CT** |  |
| --- | --- | --- | --- | --- | --- | --- | --- | --- | --- | --- | --- |
| **Facial uncleanliness measure** | **PR (95%CI)** | ***P*-value** |  | **PR (95%CI)** | ***P*-value** |  | **PR (95%CI)** | ***P*-value** |  | **PR (95%CI)** | ***P*-value** |
| Wet Nasal Discharge | 1.3 (1.1–1.5) | <0.001 |  | 1.2 (1.0–1.5) | 0.115 |  | 1.2 (1.1–1.4) | 0.001 |  | 1.3 (0.9–1.9) | 0.123 |
| Dry Nasal Discharge | 1.1 (1.0–1.3) | 0.153 |  | 1.0 (0.8–1.2) | 0.683 |  | 1.1 (1.0–1.3) | 0.058 |  | 1.1 (0.8–1.5) | 0.698 |
| Wet Ocular Discharge | 1.1 (0.8–1.5) | 0.504 |  | 1.5 (1.1–2.0) | 0.004 |  | 1.1 (0.9–1.3) | 0.601 |  | 1.0 (0.5–1.8) | 0.979 |
| Dry Ocular Discharge | 1.3 (1.1–1.5) | <0.001 |  | 1.5 (1.2–1.8) | <0.001 |  | 1.3 (1.2–1.5) | <0.001 |  | 1.9 (1.3–2.8) | 0.002 |
| Food | 1.0 (0.7–1.5) | 0.805 |  | 1.2 (0.8–1.8) | 0.353 |  | 1.1 (0.9–1.3) | 0.505 |  | 1.1 (0.5–2.1) | 0.880 |
| Dirt | 1.1 (0.9–1.3) | 0.356 |  | 1.1 (0.9–1.4) | 0.256 |  | 1.1 (1.0–1.3) | 0.100 |  | 0.9 (0.6–1.5) | 0.763 |
| Flies | 1.2 (1.1–1.5) | 0.011 |  | 1.3 (1.0–1.6) | 0.074 |  | 1.2 (1.0–1.4) | 0.010 |  | 1.2 (0.8–1.8) | 0.386 |

CT = ocular *Chlamydia trachomatis*; TF = trachomatous inflammation–follicular; TI = trachomatous inflammation–intense
